# METTL7B attenuates pathological cardiac hypertrophy via m6A-associated regulation of HK3

**DOI:** 10.1007/s11010-026-05737-1

**Published:** 2026-09-15

**Authors:** Zhen Bao, Zhi-qiang Liu, Yu-Chun Yang, Kai Wang, Muhuyati Wulasihan

**Affiliations:** 1 Postdoctoral Research Station of Public Health and Preventive Medicine, School of Public Health, Xinjiang Medical University, Urumqi, 830017 China; 2 Department of Comprehensive Cardiology, The First Affiliated Hospital of Xinjiang Medical University, Urumqi, 830054 Xinjiang China; 3 College of Medical Engineering and Technology, Xinjiang Medical University, Urumqi, 830017 Xinjiang China

**Keywords:** Pathological cardiac hypertrophy, Cardiac remodeling, METTL7B, HK3, m6A methylation, YTHDC1

## Abstract

Pathological cardiac hypertrophy is a major contributor to heart failure, yet the epitranscriptomic mechanisms that restrain maladaptive remodeling remain incompletely understood. In this study, we investigated the role of METTL7B and its association with m6A-related regulation of HK3 in pathological cardiac hypertrophy. By integrating RNA-seq and MeRIP-seq data from human hypertrophic and failing hearts, we identified coordinated downregulation of METTL7B and HK3, accompanied by reduced m6A enrichment of HK3 transcripts. Additional analysis of an independent human hypertrophic cardiomyopathy dataset showed a positive correlation between METTL7B and HK3 expression. These findings were further validated in angiotensin II-treated AC16 cardiomyocytes. Functional studies showed that overexpression of either METTL7B or HK3 attenuated cardiomyocyte hypertrophy in vitro and alleviated transverse aortic constriction-induced cardiac remodeling and dysfunction in vivo. RNA pull-down assays showed enrichment of METTL7B in HK3 RNA pull-down complexes, and METTL7B overexpression was accompanied by increased HK3 m6A enrichment and restored HK3 expression under hypertrophic stress. In vitro rescue experiments showed that HK3 silencing partially counteracted the anti-hypertrophic effect of METTL7B overexpression, supporting HK3 as a functional downstream effector of METTL7B in cardiomyocytes. We also identified YTHDC1 as a candidate HK3 RNA-associated factor. YTHDC1 knockdown stabilized HK3 mRNA, increased HK3 abundance, and reduced hypertrophic marker expression in METTL7B-overexpressing cardiomyocytes. Collectively, these findings identify a METTL7B-HK3 regulatory relationship in pathological cardiac hypertrophy and implicate YTHDC1 as a potential modulator of HK3 regulation. Our study suggests that METTL7B suppresses hypertrophic remodeling, at least in part, in association with m6A-associated regulation of HK3.

**Supplementary Information:**

The online version contains supplementary material available at https://doi.org/10.1007/s11010-026-05737-1.

## Introduction

Pathological cardiac hypertrophy is a maladaptive response of the heart to chronic hemodynamic overload and other forms of stress, including hypertension, valvular disease, myocardial ischemia, and inherited cardiomyopathies [1, 2]. Although initially compensatory, sustained hypertrophic remodeling is characterized by cardiomyocyte enlargement, interstitial fibrosis, contractile dysfunction, and profound metabolic reprogramming, which ultimately predispose the heart to failure [3–5]. Clinically, cardiac hypertrophy is not only an early hallmark of disease progression but also an independent risk factor for arrhythmia, heart failure, and cardiovascular death [6, 7]. Current therapeutic strategies, including neurohumoral blockade targeting angiotensin II (Ang II) and β-adrenergic signaling, can partially delay disease progression but remain insufficient to fully prevent maladaptive remodeling [8]. A better understanding of the molecular mechanisms that govern pathological cardiac hypertrophy is therefore essential for identifying new therapeutic targets.

N6-methyladenosine (m6A) is the most prevalent internal modification in eukaryotic messenger RNA and is increasingly recognized as an important regulator of post-transcriptional gene expression [9, 10]. Through coordinated actions of m6A writers, erasers, and readers, this reversible RNA modification controls multiple aspects of RNA metabolism, including splicing, export, translation, localization, and stability [11, 12]. In the cardiovascular system, mounting evidence suggests that aberrant m6A signaling contributes to cardiac remodeling and heart failure [13–15]. In particular, m6A reprogramming has been implicated in pathological cardiac hypertrophy, and canonical methyltransferases such as METTL3 and METTL14 have been shown to regulate hypertrophic responses through distinct downstream pathways [16–18]. However, the m6A regulatory network in the hypertrophic heart remains incompletely defined, particularly in clinically relevant settings of advanced hypertrophy and heart failure. Moreover, how epitranscriptomic regulation may intersect with metabolism-related remodeling during cardiac hypertrophy remains incompletely understood.

To identify clinically relevant m6A-associated regulators in pathological cardiac hypertrophy, we analyzed public RNA-seq and MeRIP-seq datasets derived from human hypertrophic and failing hearts. Among methyltransferase-like family members, METTL7B was identified as a significantly downregulated candidate. In parallel, analysis of the hexokinase family revealed that HK3 showed concordant decreases in both transcript abundance and m6A enrichment. These findings were of particular interest because metabolic remodeling is a defining feature of pathological hypertrophy [19], and hexokinases are key enzymes involved in glucose utilization and cellular energy homeostasis, suggesting that HK3 may have metabolism-related relevance in hypertrophic remodeling. Although METTL7B has been implicated in methylation-related processes and disease progression in other biological contexts, its role in cardiovascular remodeling remains unknown. Likewise, the contribution of HK3 to pathological cardiac hypertrophy and its potential epitranscriptomic regulation have not been established.

Based on these observations, we hypothesized that METTL7B may exert a protective role in pathological cardiac hypertrophy through regulation of HK3 expression and m6A enrichment. In the present study, we combined human dataset analysis with gain- and loss-of-function experiments in Ang II-treated cardiomyocytes and transverse aortic constriction (TAC) mouse models to investigate the role of the METTL7B-HK3 pathway in hypertrophic remodeling. We further explored the potential involvement of the m6A reader YTHDC1 in HK3 regulation. Our study identifies a previously unrecognized METTL7B-HK3 regulatory relationship and provides new insight into how epitranscriptomic control may modulate pathological cardiac hypertrophy.

## Materials and methods

### Human public dataset acquisition and analysis

Publicly available transcriptomic and epitranscriptomic data were obtained from the Gene Expression Omnibus database. The GSE159243 dataset, generated on platform GPL11154, was used for the analysis of pathological cardiac hypertrophy and heart failure. This dataset included 20 human heart samples, comprising 10 disease samples and 10 control samples. Among them, RNA sequencing data were available for 5 samples in each group, whereas the remaining samples were subjected to methylated RNA immunoprecipitation sequencing for m6A profiling. The disease group consisted of myocardial samples from patients with left ventricular hypertrophy and nonischemic heart failure, while control samples were obtained from healthy donor hearts without a history of heart failure [14]. Expression changes in methyltransferase-related genes and hexokinase family members, together with m6A modification changes, were analyzed to identify candidate regulators associated with pathological cardiac hypertrophy. Differential expression analysis was performed using normalized gene expression matrices obtained from GEO. Genes with *P* < 0.05 and the indicated fold-change threshold were considered differentially expressed. Candidate genes were selected based on concordant changes in transcript abundance and m6A enrichment.

To independently validate the clinical relevance of the METTL7B-HK3 association, an additional publicly available human hypertrophic cardiomyopathy transcriptomic dataset, GSE180313, was analyzed. This validation dataset included 20 human myocardial samples, comprising 7 normal control samples and 13 hypertrophic cardiomyopathy samples. METTL7B and HK3 expression levels were extracted from the normalized expression matrix and presented as FPKM values. Differential expression of METTL7B and HK3 between hypertrophic cardiomyopathy and control samples was evaluated, and adjusted P values were used where available to account for multiple testing. Pearson correlation analysis was performed to assess the association between METTL7B and HK3 expression in control and hypertrophic cardiomyopathy samples separately.

### Reagents and antibodies

Antibodies against atrial natriuretic peptide (ANP) (sc-515701, Texas, USA), brain natriuretic peptide (BNP) (sc-271185, Texas, USA), and β-myosin heavy chain (β-MHC) (sc-53090, Texas, USA) were purchased from Santa Cruz Biotechnology. Anti-METTL7B antibody (ab110134, Cambridge, UK) and horseradish peroxidase-conjugated secondary antibodies (goat anti-rabbit IgG H&L, ab205718; goat anti-mouse IgG H&L, ab205719, Cambridge, UK) were purchased from Abcam. Anti-HK3 polyclonal antibody (PA5-121197, USA) was purchased from Thermo Fisher Scientific. Anti-YTHDC1 polyclonal antibody (14392-1-AP, Chicago, USA) was obtained from Proteintech. Anti-β-actin antibody (100166-MM10, Beijing, China) was purchased from Sino Biological. Ang II (1158, Bristol, UK) was obtained from Tocris Bioscience, and the prestained protein marker (PR1910, Beijing, China) was purchased from Solarbio.

### Cell culture and establishment of the hypertrophic cardiomyocyte model

The human cardiomyocyte cell line AC16 (YS1012C, Shanghai, China) was obtained from YaJi Biosciences and cultured in high-glucose DMEM (GIBCO, C11995500BT, Massachusetts, USA) supplemented with 10% fetal bovine serum (ExCell Bio, FND500, Shanghai, China) and 1% penicillin-streptomycin (GIBCO, 15070-063, Massachusetts, USA) at 37 °C in a humidified incubator containing 5% CO2. Cells were routinely tested and confirmed to be free of mycoplasma contamination before use. To establish the hypertrophic model, AC16 cells were treated with 200 nM Ang II for 24 h, whereas control cells were cultured in parallel without Ang II stimulation. Cells were subsequently collected for RNA, protein, or morphological analyses.

### siRNA transfection and lentiviral overexpression in AC16 cells

For siRNA transfection, AC16 cells were seeded into 24-well plates at a density of 5 × 10^4 cells per mL and cultured for 24 h before transfection. siRNAs were transfected using Lipofectamine RNAiMAX (Thermo Fisher, 13778-150, USA) according to the manufacturer’s instructions. Briefly, siRNA and transfection reagent were diluted separately, mixed to allow complex formation for 10 min at room temperature, and then added to cells in fresh medium. After 6 h, the transfection medium was replaced with complete medium. Transfection efficiency was initially evaluated using Block-iT Alexa Fluor Red oligonucleotides (Thermo Fisher, 14750-100, USA) by fluorescence microscopy at 24 and 48 h. A scrambled siRNA served as the negative control. The most efficient siRNA sequence was then selected based on quantitative real-time polymerase chain reaction (PCR), and knockdown efficiency at the protein level was confirmed by western blotting. Finally, a siRNA concentration of 0.1 µM and a transfection time of 48 h were selected as the optimal conditions for subsequent experiments. SiRNAs targeting METTL7B, HK3, and YTHDC1 were synthesized by General Biosystems, Anhui, China. The siRNA sequences are provided in Table 1.

**Table 1:** siRNA primer information sheet

Primer name	Primer sequence (5′ to 3′)
hsa-METTL7B (ID:196410) siRNA-736	GGGAAAGGCUGUCAAAUAATT
	UUAUUUGACAGCCUUUCCCTT
hsa-METTL7B (ID:196410) siRNA-162	ACCGCAAGAUGGAGAGCAATT
	UUGCUCUCCAUCUUGCGGUTT
hsa-METTL7B (ID:196410) siRNA-342	CAAAGAGCAUGGCUGAGAATT
	UUCUCAGCCAUGCUCUUUGTT
hsa-HK3 (ID:3101) siRNA-237	GGGCACAGCUACAGCAGAUTT
	AUCUGCUGUAGCUGUGCCCTT
hsa-HK3 (ID:3101) siRNA-1610	GCACAGAUGCGGAAGGCCATT
	UGGCCUUCCGCAUCUGUGCTT
hsa-HK3 (ID:3101) siRNA-805	GGUCAGGCCGUGUGAGGUUTT
	AACCUCACACGGCCUGACCTT
siRNA-NC	UUCUCCGAACGUGUCACGUTT
	ACGUGACACGUUCGGAGAATT
hsa-YTHDC1 NM_001031732.4 -siRNA-1240	GAAGAGAGCUAGAGGCAUATT
	UAUGCCUCUAGCUCUCUUCTT
hsa-YTHDC1 NM_001031732.4 -siRNA-358	GGAGAAAGAUGGAGAACUUTT
	AAGUUCUCCAUCUUUCUCCTT
hsa-YTHDC1 NM_001031732.4 -siRNA-2270	GAAGCAAGAUACAGAGAUATT
	UAUCUCUGUAUCUUGCUUCTT

For lentiviral transduction, AC16 cells in logarithmic growth phase were seeded into 6-well plates at a density of 5 × 10^4 cells per mL. When cells reached approximately 60% confluence, the medium was replaced with fresh medium containing recombinant lentivirus and transduction enhancer (Hitrans G P, GeneChem, Shanghai, China). After 12 h, the medium was changed to complete medium and the cells were cultured for an additional 72 h. Transduction efficiency was monitored by fluorescence microscopy, and only cultures showing high fluorescence intensity, approximately 80% transduction efficiency, and preserved cellular morphology were used for subsequent experiments. Overexpression efficiency was verified by quantitative real-time PCR and western blotting. Empty vector-transduced cells and nontransduced cells were used as controls where appropriate. The optimal infection conditions were determined as follows: AC16 cells were transduced at a multiplicity of infection (MOI) of 10 using lentivirus at a titer of 1 × 10⁷ TU/mL. The transduction mixture consisted of 86 µL of regular culture medium, 10 µL of virus stock, and 4 µL of Hitrans G P. Lentiviral vectors encoding METTL7B, HK3, or NC were obtained from GeneChem, Shanghai, China.

### Wheat germ agglutinin (WGA) staining of cultured cardiomyocytes

For assessment of cardiomyocyte size, AC16 cells were seeded into 24-well plates at a density of 5 × 10^4 cells per mL and allowed to adhere overnight. Cells were then treated with 200 nM Ang II for 24 h or left untreated as controls. After treatment, cells were washed with phosphate-buffered saline and incubated with iFluor 488-conjugated WGA (Solarbio, I3300, Beijing, China) at a final concentration of 10 µg per mL for 30 min at 37 °C in the dark. Cells were washed again with phosphate-buffered saline and imaged under a fluorescence microscope. Cell surface area was quantified from WGA-stained images.

### Immunofluorescence staining

For immunofluorescence analysis, AC16 cells were adjusted to a density of 1 × 10^5 cells per mL and seeded onto adhesive microscope slides in complete medium. After 24 h, cells were subjected to the indicated treatments. Cells were then fixed in 4% paraformaldehyde for 10 min, permeabilized with 0.5% Triton X-100 for 20 min, and blocked with 1% bovine serum albumin for 40 min at room temperature. Samples were incubated overnight at 4 °C with anti-α-actinin primary antibody (Santa Cruz Biotechnology, sc-17829, Texas, USA), washed, and then incubated with Alexa Fluor 488-conjugated goat anti-mouse IgG (Abcam, ab150113, Cambridge, UK) for 1 h at 37 °C in the dark. Nuclei were counterstained with DAPI (Shanghai Fusheng Biotechnology, FSM0180-100T, Shanghai, China), and slides were mounted with 50% glycerol. Fluorescence images were acquired using a confocal laser scanning microscope (Leica TCS SP8, Leica Microsystems, Wetzlar, Germany). Cardiomyocyte area was quantified from α-actinin-positive cells in randomly selected fields.

### Animal experiments

All animal procedures were approved by the Animal Ethics Committee of the First Affiliated Hospital of Xinjiang Medical University and were performed in accordance with the Guide for the Care and Use of Laboratory Animals. The approval number was 250303-309. A total of 80 male specific pathogen-free C57BL/6 mice aged 10 weeks and weighing 25 to 28 g were purchased from Hangzhou Qizhen Experimental Animal Technology Co., Ltd. The animal quality certificate number was 20250418Abzz01009990023, and the production and use license numbers were SCXK (Zhejiang) 2022-0005 and SYXK (Zhejiang) 2023-0045, respectively. Of these, 60 mice were included in the formal in vivo experiment and randomly assigned to five groups, with 12 mice per group. The sample size for the formal animal experiment was determined based on commonly used sample sizes in TAC mouse studies, anticipated perioperative attrition, experimental feasibility, and animal welfare considerations. The remaining 20 mice were maintained as reserve animals to avoid insufficient group size due to potential loss during acclimatization, housing, or surgical procedures, and were not included in the experimental groups or statistical analyses. Mice were housed under standard laboratory conditions at 22 ± 2 °C with 60 to 80% relative humidity and a regular light-dark cycle, with free access to food and water. Animals were randomly assigned to the indicated groups, and surgical procedures and data analyses were performed in a blinded manner. For all in vivo analyses, each mouse was considered an independent biological replicate, and the exact number of biological replicates used for each assay is indicated in the corresponding figure legends.

### AAV9 delivery and experimental design

Recombinant cardiotropic adeno-associated virus serotype 9 vectors encoding METTL7B, HK3, or the corresponding negative control were purchased from Shanghai GeneChem Co., Ltd. The negative control vector was an AAV empty vector. To achieve in vivo overexpression, mice received a single tail vein injection of AAV two weeks before transverse aortic constriction surgery. Each AAV-treated mouse received 1 × 10¹¹ viral genomes in 100 µL sterile saline, corresponding to a final working concentration of 1 × 10¹² vg/mL. To obtain the same final injection dose, different volumes of viral stocks were diluted with sterile saline. Specifically, 52 µL of NC AAV stock was diluted with 2248 µL saline, 63 µL of AAV-METTL7B stock was diluted with 2237 µL saline, and 142 µL of AAV-HK3 stock was diluted with 2158 µL saline. The diluted viral solutions were prepared immediately before injection and kept on ice.

The 60 mice included in the formal experiment were randomly assigned to five groups, with 12 mice per group: sham, TAC, TAC plus NC AAV, TAC plus AAV-METTL7B, and TAC plus AAV-HK3. Mice in the sham and TAC groups received sterile saline, whereas mice in the AAV-treated groups received the corresponding AAV vector two weeks before TAC surgery. Sham-operated mice underwent the same surgical procedure as TAC mice except that the transverse aorta was not ligated. All mice were sacrificed on day 20 after TAC or sham surgery for subsequent analyses.

### TAC model

Pathological cardiac hypertrophy was induced by TAC as previously described [20]. Briefly, mice were fasted for 12 h before surgery. Mice anesthetized with isoflurane were carefully placed on the platform and connected to a non-invasive ventilator for assisted ventilation. After removal of chest hair and skin disinfection, a midline cervical incision was made. The thyroid gland was retracted, the trachea was exposed, and a median sternotomy was performed to visualize the aortic arch. Surrounding connective tissue was gently separated, and a 7 − 0 silk suture was tied around the transverse aorta against a 27-gauge needle. The suture was tightened and knotted, after which the needle was removed to produce standardized constriction of the aorta. Sham-operated mice underwent the same surgical procedure except that the aorta was not ligated. Body temperature was maintained at 37 °C throughout surgery and recovery. After surgery, the thoracic cavity was cleared of residual fluid, the chest and skin were closed in layers, and mice were returned to their cages after recovery from anesthesia.

### Echocardiographic assessment

Echocardiography was performed on day 20 after TAC or sham surgery before sacrifice. Mice were anesthetized and positioned on the examination platform. Cardiac function was assessed using a VINNO 6 LAB small animal ultrasound imaging system (Vinno Technology Co., Ltd., Suzhou, China). The following parameters were measured according to standard procedures: heart rate, fractional shortening (FS), ejection fraction (EF), left ventricular end-diastolic volume, left ventricular end-systolic volume, and left ventricular chamber dimensions and wall thicknesses during systole and diastole. All echocardiographic measurements were performed in a blinded manner.

### Tissue collection and morphometric analysis

On day 20 after TAC surgery, mice were anesthetized and euthanized for sample collection. Blood samples were collected via retro-orbital bleeding under deep anesthesia, allowed to clot for 30 min, and centrifuged at 2000 × g for 15 min to obtain serum, which was stored at − 80 °C. Whole hearts from 6 mice per group were excised, washed, blotted dry, weighed, photographed, and fixed in 4% paraformaldehyde for histological analysis. Left ventricles from the remaining animals were dissected, weighed, snap-frozen in liquid nitrogen, and stored at − 80 °C for molecular analyses. Heart weight-to-body weight ratio (HW/BW) was calculated for each mouse, and left ventricular mass index (LVMI) was calculated as left ventricular weight in mg divided by body weight in g.

### Histological staining and analysis

For histological examination, myocardial tissues were fixed in 4% paraformaldehyde, embedded in paraffin, and sectioned for staining. Hematoxylin and eosin (H&E) staining (Biosharp, BL735B-1/BL735B-2, Hefei, China) was performed to evaluate myocardial structural changes and cardiomyocyte hypertrophy. Masson’s trichrome staining (Biosharp, BL1538A, Hefei, China) was used to assess interstitial and perivascular fibrosis. For cardiomyocyte cross-sectional area analysis, transverse sections from the mid-left ventricle were stained with fluorescently conjugated WGA to delineate cell membranes. Images were acquired from randomly selected left ventricular myocardial fields under identical imaging settings. Fields containing large vessels or extensive perivascular structures were avoided. Cardiomyocyte cross-sectional area was measured using ImageJ. Only transversely sectioned cardiomyocytes showing a complete and continuous WGA-positive sarcolemmal boundary and a round or polygonal morphology were included. Vascular lumens, endothelial structures, perivascular regions, interstitial WGA-positive signals, and obliquely or longitudinally sectioned myocytes were excluded. Quantification was performed by investigators blinded to the experimental groups.

### RNA extraction and quantitative real-time PCR

Total RNA was extracted from AC16 cells or myocardial tissue using TRIzol reagent (Ambion, 15596026, Austin, USA) according to the manufacturer’s instructions. RNA concentration and purity were assessed by microspectrophotometry, and RNA integrity was verified by agarose gel electrophoresis. First-strand cDNA was synthesized from 1 µg total RNA using a commercial reverse transcription kit (Applied Biological Materials, G492, Richmond, Canada) under RNase-free conditions. Quantitative real-time PCR was performed using 2 × SYBR Green qPCR Master Mix (Servicebio, G3321-15, Wuhan, China) with gene-specific primers on a real-time PCR system. A melting curve analysis was performed after amplification to verify product specificity. Target gene expression was normalized to β-actin using the 2 − ΔΔCt method. All primers were synthesized by Sangon Biotech, Shanghai, China. Primer sequences used for quantitative PCR are listed in Table 2.

**Table 2:** Quantitative real-time PCR mRNA primer information sheet

Primer name	Primer sequence (5′ to 3′)	Amplicon size
METTL7B-F	ATGTGGTGGTCTGCACTCTG	166 bp
METTL7B-R	GTGGGCTCGAAAACTTGCTG	
β-actin-F	GGACTTCGAGCAAGAGATGG	234 bp
β-actin-R	AGCACTGTGTTGGCGTACAG	
HK3-F	GTGAGGTTGGGCTAGTTGTAGA	176 bp
HK3-R	GTCCAGGGTATGGTCGAAGGT	
YTHDC1-F	ACAACCTCCACACCATCCTT	176 bp
YTHDC1-R	CTTGTGTGCGACGAAGGAAA	

### Protein extraction and western blot analysis

AC16 cells or myocardial tissues were lysed on ice for 60 min in RIPA lysis buffer (Boster, AR0105, Wuhan, China) supplemented with protease inhibitor cocktail (Boster, AR1178, Wuhan, China). Lysates were centrifuged, and the supernatants were collected as total protein extracts. Protein concentrations were determined using a bicinchoninic acid protein assay kit (TransGen Biotech, DQ111-01, Beijing, China). Equal amounts of protein, typically 30 µg per lane, were separated by sodium dodecyl sulfate-polyacrylamide gel electrophoresis and transferred onto polyvinylidene fluoride membranes (Merck Millipore, IPVH00010 and ISEQ00010, Billerica, USA). Membranes were blocked with 5% nonfat milk for 1 h at room temperature and incubated overnight at 4 °C with primary antibodies against the indicated proteins. After washing, membranes were incubated with horseradish peroxidase-conjugated secondary antibodies for 1 h at room temperature. Protein bands were visualized using enhanced chemiluminescence reagent (Proteintech, PK10003, Wuhan, China), and β-actin was used as the loading control. Band intensities were quantified by densitometry where indicated. Protein levels were normalized to β-actin as indicated.

### Methylated RNA immunoprecipitation followed by quantitative PCR (MeRIP-qPCR)

The m6A modification level of HK3 mRNA was determined by methylated RNA immunoprecipitation followed by quantitative PCR. Briefly, total RNA was isolated from control and Ang II-treated AC16 cells using TRIzol reagent. Polyadenylated mRNA was enriched using oligo dT-conjugated magnetic beads (Thermo Fisher, 25–61005, USA) and fragmented using a magnesium-based RNA fragmentation module (New England Biolabs, E6150S, Ipswich, USA). Fragmented mRNA was incubated with m6A antibody (Synaptic Systems, 202003, Göttingen, Germany) conjugated magnetic beads (Thermo Fisher, Dynabeads Antibody Coupling Kit, 14311D, California, USA) in IP buffer (50 mM Tris-HCl, 750 mM NaCl, 0.5% Igepal CA-630) for immunoprecipitation. After washing, bound RNA was eluted and purified. IP-enriched RNA was reverse-transcribed into cDNA using SuperScript™ II Reverse Transcriptase (Invitrogen, 1896649, California, USA). Input, IgG control, and m6A immunoprecipitated fractions were subjected to quantitative PCR to determine HK3 transcript enrichment. Relative m6A enrichment was calculated by comparison of the m6A immunoprecipitated fraction with the corresponding input fraction.

### RNA pull-down assay and mass spectrometry

For the RNA pull-down assay, the probe template corresponding to the HK3 mRNA sequence was generated from a chemically synthesized DNA fragment; the full HK3 mRNA sequence is provided in the Supplementary File. Three pairs of primers were designed according to the human HK3 mRNA sequence to amplify three overlapping PCR fragments covering the full-length coding region of HK3. The primer sequences are listed in Table 3. PCR amplification was performed using an HK3-containing plasmid as the template and upstream primers containing T7, SP6, or T3 promoter sequences. PCR products were purified with AMPure XP Nucleic Acid Purification Kit (Beckman Coulter, A63882, California, USA) and quantified using a Qubit 4.0 fluorometer. Equal amounts of the three purified fragments were pooled as templates for in vitro transcription using the corresponding MEGAscript transcription kits according to the manufacturer’s instructions (Invitrogen, AM1334, AM1330, and AM1338, Austin, TX, USA). HK3 RNA transcripts were purified with the RNAClean XP kit (Beckman Coulter, A63987, Indianapolis, USA), quantified, and biotinylated at the 3′ end using the Pierce RNA 3′ End Biotinylation Kit (Thermo Fisher, 20160, USA). For pull-down, streptavidin magnetic beads were equilibrated in 2 × B&W buffer containing 10 mM Tris-HCl, pH 7.5, 1 mM EDTA, and 2 M NaCl, and incubated with 1 µg biotin-labeled HK3 RNA for 15 min at room temperature. Bead-RNA complexes were washed three times with 1× B&W buffer. AC16 cells were lysed on ice for 10 min in lysis buffer containing 20 mM HEPES-KOH, pH 7.3, 10 mM KCl, 10 mM NaCl, 0.5% NP-40, 10% glycerol, and 2 mM MgCl_2_, supplemented with protease and RNase inhibitors. Lysates were centrifuged at 16,000 × g for 10 min at 4 °C, diluted with four volumes of DPB binding buffer, and incubated with bead-RNA complexes overnight at 4 °C. The beads were washed five times with DPB buffer (20 mM HEPES-KOH, pH 7.3, 10 mM KCl, 10 mM NaCl, 10% glycerol, 2 mM MgCl_2_, proteinase inhibitor, RNase inhibitor), and bound proteins were eluted by boiling in 100 µL of 2 × SDS loading buffer at 100 °C for 5 min for subsequent western blotting or mass spectrometry. Mass spectrometry analysis was performed by Xinyao Biotechnology, Hangzhou, China, and candidate proteins were ranked according to spectral intensity.

**Table 3:** Primers for in vitro transcription of RNA in RNA pulldown assay

T7HK3F1	ATCGATCTCGATCCCGCGA GCTTTTGGTCATTGGGGTGC
SP6HK3F1	ATTTAGGTGACACTATA GCTTTTGGTCATTGGGGTGC
T3HK3F1	AATTAACCCTCACTAAAG GCTTTTGGTCATTGGGGTGC
HK3R1	ttGCGCCCCATCATCGCTGA
T7HK3F2	TAATACGACTCACTATAGGGAGACGTGTGAGGTTGGGCTAGT
SP6HK3F2	ATTTAGGTGACACTATAGAAGaGCGTGTGAGGTTGGGCTAGT
T3HK3F2	AATTAACCCTCACTAAAGGGAGACGTGTGAGGTTGGGCTAGT
HK3R2	ttGCTGCCTACATGGGAAGGAG
T7HK3F3	TAATACGACTCACTATAGGGAGAGGACTTCCAGCAGAAGCAGG
SP6HK3F3	ATTTAGGTGACACTATAGAAGaGGGACTTCCAGCAGAAGCAGG
T3HK3F3	AATTAACCCTCACTAAAGGGAGAGGACTTCCAGCAGAAGCAGG
HK3R3	ttAACCTGAGTGCTACTTCTCTCAGG

### RNA immunoprecipitation-qPCR (RIP-qPCR)

RNA immunoprecipitation followed by qPCR was performed to determine whether YTHDC1 was associated with HK3 mRNA in AC16 cardiomyocytes. AC16 cells were harvested under RNase-free conditions and lysed in RIP lysis buffer supplemented with protease inhibitors and RNase inhibitor. A portion of the cell lysate was saved as the input control, and the remaining lysate was incubated with magnetic protein A/G beads coupled to an anti-YTHDC1 antibody or normal IgG at 4 °C with gentle rotation. After incubation, the beads were washed thoroughly, and the RNA-protein complexes were dissociated. The immunoprecipitated RNA was then purified and reverse-transcribed into cDNA. HK3 mRNA levels in the input, IgG control, and YTHDC1-RIP fractions were measured by qRT-PCR. HK3 mRNA enrichment was calculated relative to the corresponding input RNA and compared between the IgG and YTHDC1-RIP fractions. RIP-qPCR experiments were performed in three independent biological replicates.

### Actinomycin D chase assay

HK3 mRNA stability was assessed using an Actinomycin D (MedChemExpress, HY-17559, USA) chase assay. Cells transfected with si-NC or si-YTHDC1 were treated with Actinomycin D at 5 µg/mL to block nascent RNA synthesis. Total RNA was extracted at 0, 2, 4, and 6 h after treatment, followed by reverse transcription and RT-qPCR analysis. HK3 mRNA expression was normalized to β-actin and then to the corresponding 0 h level in each group. The percentage of HK3 mRNA remaining was plotted over time. The apparent mRNA half-life was calculated by fitting the normalized decay data to a first-order exponential decay model, Y(t)=Y_0_e^− *kt*^, where *k* is the decay rate constant. The half-life was determined as t_1/2_=ln2/*k*. Experiments were performed in three biological replicates.

### Statistical analysis

All data are presented as mean ± standard deviation. Statistical analyses were performed using SPSS version 25.0, and graphs were generated using GraphPad Prism version 5.0. For animal experiments, individual mice were used as the statistical units. For cell-based and molecular assays, independent biological replicates were used as the statistical units unless otherwise stated. Technical replicates, when performed, were averaged before statistical analysis and were not treated as independent biological replicates. The exact sample size for each experiment is provided in the corresponding figure legends. Normality was assessed using the Shapiro-Wilk test, and homogeneity of variance was examined by Levene’s test. Comparisons between two groups were performed using an unpaired two-tailed Student’s t test when the assumptions of normality and equal variance were satisfied. Comparisons among more than two groups were analyzed by one-way analysis of variance followed by Tukey’s post hoc test when the assumption of homogeneity of variance was satisfied. When equal variance was not assumed, the Games-Howell test was used for multiple comparisons. For Actinomycin D chase assays, HK3 mRNA remaining was analyzed using two-way ANOVA to assess the effects of group, time, and their interaction. Apparent mRNA half-life was estimated by fitting normalized mRNA decay curves to a first-order exponential decay model. A value of *P* less than 0.05 was considered statistically significant.

## Results

### A suppressed METTL7B-HK3 axis with reduced HK3 m6A enrichment is associated with pathological cardiac hypertrophy

**Fig. 1:**
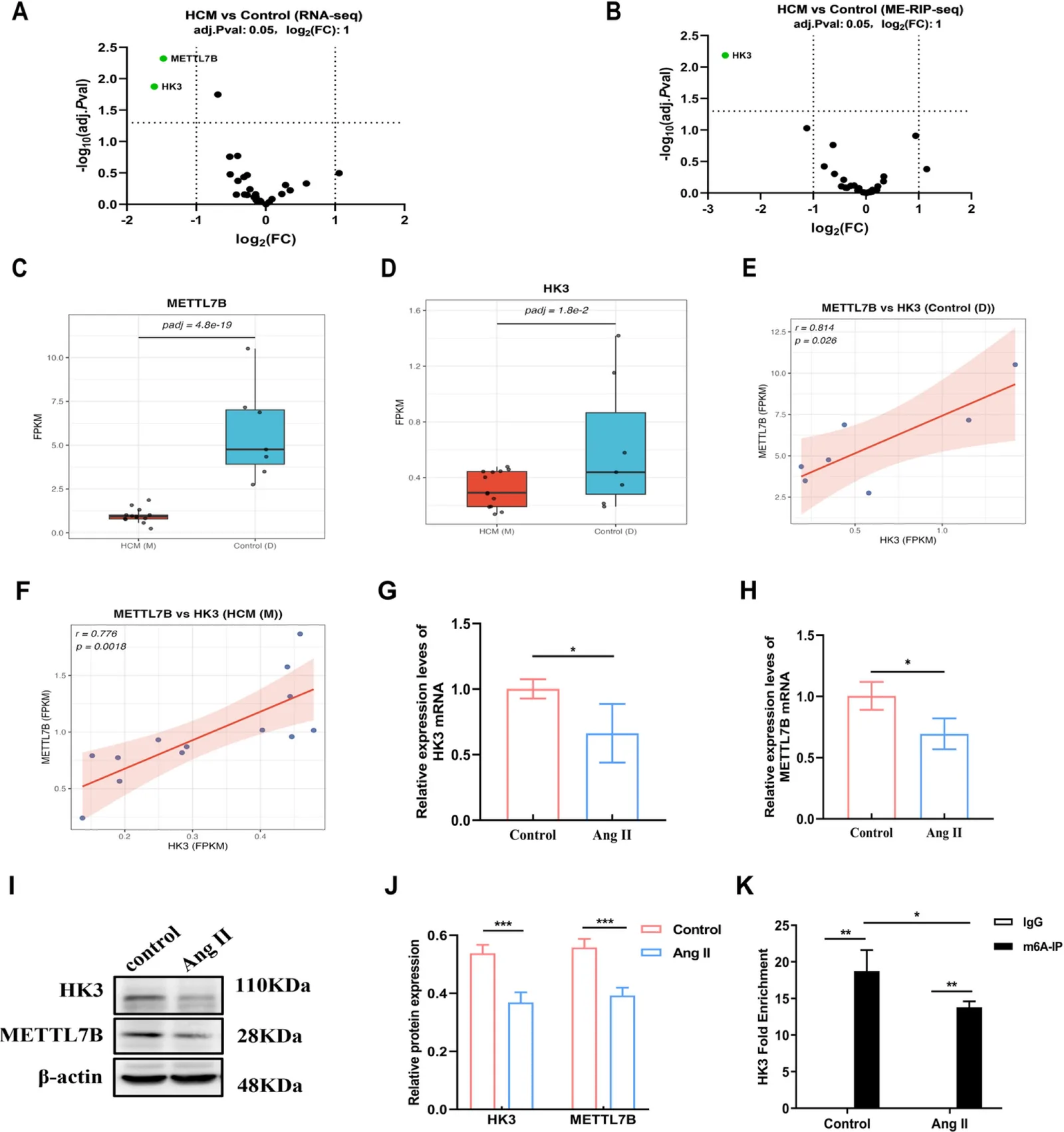
METTL7B and HK3 are downregulated in cardiac hypertrophy, accompanied by reduced HK3 m6A enrichment. **A**,** B** Volcano plots showing differentially expressed genes identified from the GSE159243 dataset by integrated RNA-seq (**A**) and methylated RNA immunoprecipitation sequencing analyses (**B**). Black dots indicate genes with no significant change, whereas green dots indicate significantly downregulated genes. **C** Boxplot showing METTL7B expression levels in HCM and control samples from the GSE180313 dataset. **D** Boxplot showing HK3 expression levels in HCM and control samples from the GSE180313 dataset. Adjusted P values are shown in the boxplots. **E** Correlation analysis of METTL7B and HK3 expression in control samples from the GSE180313 dataset. **F** Correlation analysis of METTL7B and HK3 expression in HCM samples from the GSE180313 dataset. Correlation coefficients and nominal P values are shown in the scatter plots. For panels **C**–**F**, expression values are shown as FPKM. In panels **C**–**F**, **D** denotes donor control and M denotes myectomy sample; HCM, hypertrophic cardiomyopathy. **G**,** H** qRT-PCR validation of HK3 (**G**) and METTL7B (**H**) mRNA expression levels in control and angiotensin II (Ang II)-treated AC16 cardiomyocytes. Relative mRNA levels were normalized to β-actin (*n* = 3). **I** Representative Western blot images of HK3 and METTL7B expression in control and Ang II-treated AC16 cardiomyocytes. **J** Densitometric analysis of HK3 and METTL7B protein levels in control and Ang II-treated AC16 cardiomyocytes. Protein expression was normalized to β-actin (*n* = 4). **K** MeRIP-qPCR analysis of m6A enrichment of HK3 mRNA in control and Ang II-treated AC16 cardiomyocytes (*n* = 3). For **G**, **H**, **J**, and **K**, data are presented as mean ± SD. Unless otherwise indicated, n denotes independent biological replicates. ∗*P* < 0.05, ∗∗*P* < 0.01, ∗∗∗*P* < 0.001

To identify m6A-related regulators involved in pathological cardiac hypertrophy, we performed an integrated analysis of RNA-seq and MeRIP-seq data from the GEO dataset GSE159243, which contains human cardiac tissue samples from healthy controls and patients with cardiac hypertrophy and heart failure. Among members of the methyltransferase-like family, METTL7B was the only gene significantly downregulated in hypertrophic hearts. Among the hexokinase family, HK3 was the only member showing both reduced mRNA expression and decreased m6A enrichment, whereas no significant changes were observed in other family members Fig. 1A and B.

To further validate the clinical relevance of METTL7B and HK3 in human hypertrophic cardiomyopathy, we analyzed an independent publicly available human HCM transcriptomic dataset, GSE180313. In this validation cohort, METTL7B expression was significantly reduced in HCM samples compared with control samples (adjusted *P* = 4.8 × 10⁻¹⁹, Fig. 1C). HK3 expression was also significantly reduced in HCM samples compared with controls (adjusted *P* = 1.8 × 10⁻², Fig. 1D). We next examined the relationship between METTL7B and HK3 expression in this independent cohort. METTL7B expression was positively correlated with HK3 expression in control samples (*r* = 0.814, *P* = 0.026, Fig. 1E). A significant positive correlation between METTL7B and HK3 was also observed in HCM samples (*r* = 0.776, *P* = 0.0018, Fig. 1F). Together, these findings provide independent transcriptomic validation that METTL7B and HK3 are downregulated in human HCM and that their expression levels are positively associated in human cardiac samples.

To validate these observations in vitro, we established an Ang II-induced hypertrophic model in AC16 cardiomyocytes. Consistent with the human transcriptomic data, both METTL7B and HK3 were markedly downregulated in Ang II-treated cells Fig. 1G-J. MeRIP-qPCR further showed that the m6A enrichment of HK3 mRNA was significantly decreased in hypertrophic cardiomyocytes Fig. 1K. Together, these data indicate that pathological cardiomyocyte hypertrophy is accompanied by coordinated suppression of METTL7B and HK3, together with reduced m6A enrichment of HK3 transcripts.

To further explore the potential m6A modification landscape of HK3, we performed an in silico prediction using the SRAMP tool with the RefSeq transcript NM_002115.3 as input. The analysis predicted six putative m6A sites within the HK3 transcript, located at positions 111, 1218, 1863, 1875, 1977, and 2220. Among these, the site at position 2220 showed the highest combined score (0.658) and was classified as a high-confidence m6A site (Fig. S1). Together with the MeRIP-qPCR results showing altered m6A enrichment of HK3 under hypertrophic stress, these predicted sites provide candidate motifs for future site-specific functional studies.

### METTL7B attenuates cardiomyocyte hypertrophy and is associated with restored HK3 expression and m6A enrichment

**Fig. 2:**
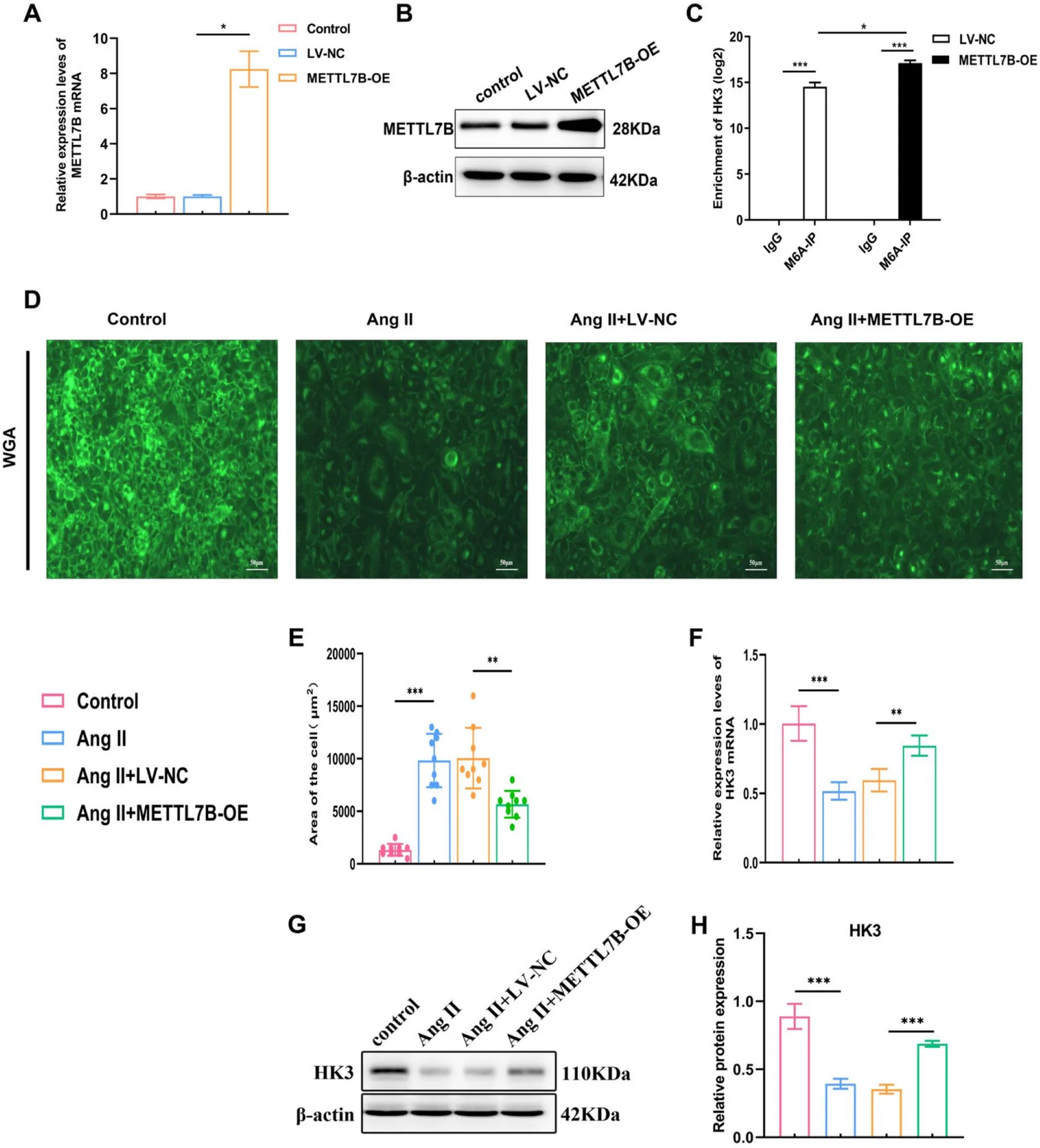
METTL7B overexpression attenuates cardiomyocyte hypertrophy and is accompanied by increased HK3 m6A enrichment. **A**, **B** Validation of METTL7B overexpression in AC16 cardiomyocytes by qRT-PCR (**A**) and western blotting (**B**). Relative mRNA levels were normalized to β-actin. Protein expression was normalized to β-actin (*n* = 3). **C** MeRIP-qPCR analysis of m6A enrichment of HK3 mRNA in control and METTL7B-overexpressing AC16 cardiomyocytes (*n* = 3). **D** Representative wheat germ agglutinin (WGA) staining images of AC16 cardiomyocytes. Scale bar,  50 μm. **E** Quantification of cell area in panel **D** (*n* = 9). **F–H** Analysis of HK3 expression, including qRT-PCR analysis of HK3 mRNA levels (**F**), representative western blot images (**G**), and densitometric quantification of HK3 protein expression (**H**). Relative mRNA levels were normalized to β-actin. Protein expression was normalized to β-actin (*n* = 3). Data are presented as mean ± SD. Unless otherwise indicated, n denotes independent biological replicates. ∗*P* < 0.05, ∗∗*P* < 0.01, ∗∗∗*P* < 0.001

Given the concomitant reduction of METTL7B expression and HK3 m6A abundance in hypertrophic cardiomyocytes, we next asked whether METTL7B regulates HK3 and contributes to the hypertrophic phenotype. Lentiviral METTL7B overexpression was validated in AC16 cells. METTL7B mRNA expression was markedly increased after lentiviral METTL7B transduction Fig. 2A. Western blot analysis further showed an increase in METTL7B protein abundance in METTL7B-overexpressing cells Fig. 2B. MeRIP-qPCR analysis demonstrated that METTL7B overexpression was associated with significantly increased m6A enrichment of HK3 mRNA Fig. 2C, suggesting that METTL7B is linked to altered HK3 m6A enrichment under hypertrophic stress.

Functionally, WGA staining showed that METTL7B overexpression markedly attenuated Ang II-induced cardiomyocyte enlargement Fig. 2D and E. In parallel, METTL7B overexpression restored HK3 expression at both the mRNA and protein levels under hypertrophic conditions Fig. 2F-H. These results indicate that METTL7B acts as an endogenous suppressor of cardiomyocyte hypertrophy and suggest that its protective effect is associated with restoration of HK3 expression and increased HK3 m6A enrichment.

### HK3 may contribute to the anti-hypertrophic effects associated with METTL7B in vitro

To determine whether HK3 functions downstream of METTL7B, we established both gain-of-function and loss-of-function models for METTL7B and HK3 in cardiomyocytes. Efficient knockdown of METTL7B and HK3 by specific siRNAs was confirmed Fig. S2A-F, and lentiviral HK3 overexpression constructs were validated Fig. S2G-I.

**Fig. 3:**
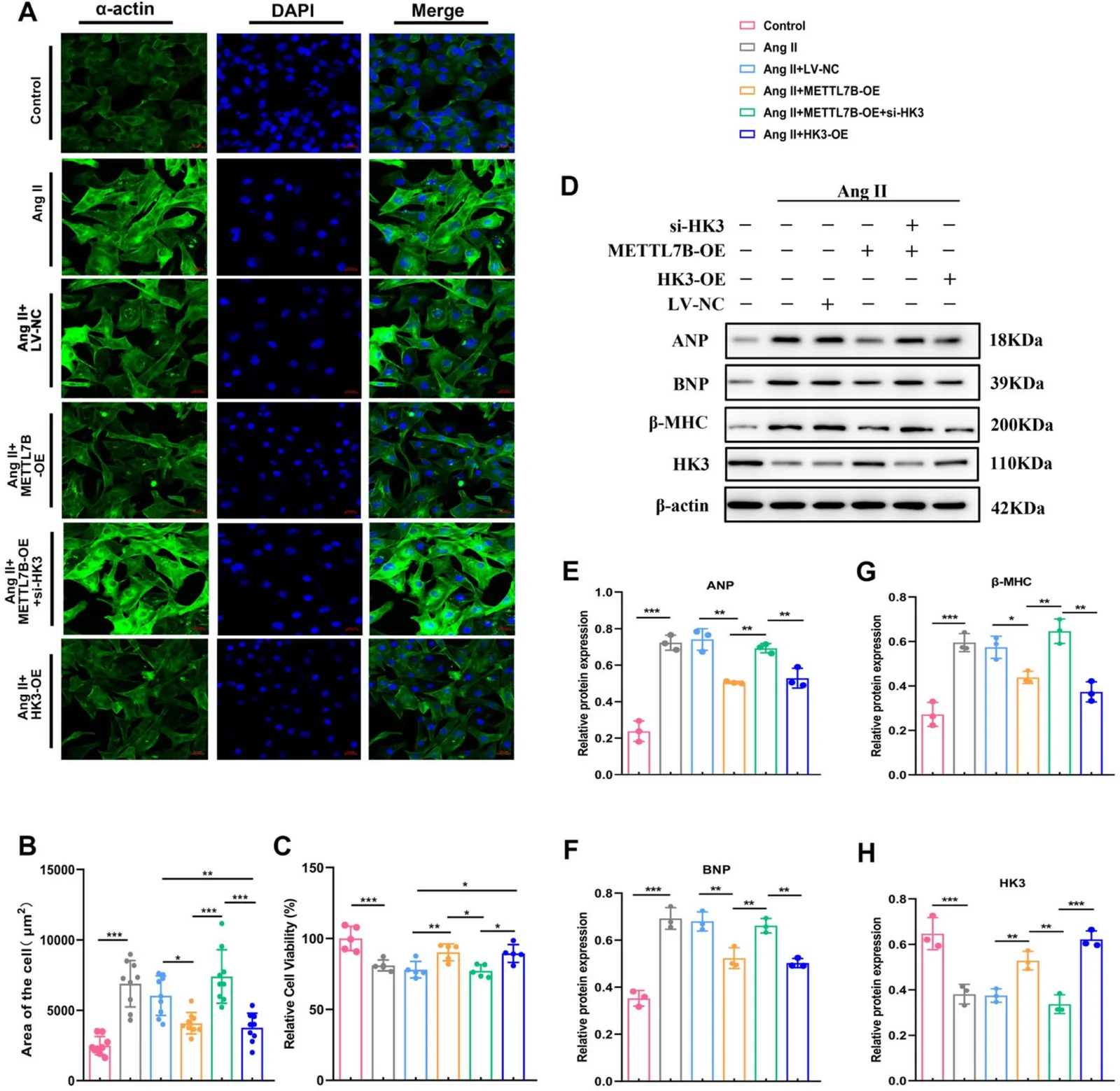
METTL7B overexpression alleviates cardiomyocyte hypertrophy in an HK3-dependent manner. **A** Representative immunofluorescence images of AC16 cardiomyocytes stained for α-actinin green, with nuclei counterstained with DAPI blue. Scale bar, 20 μm. **B** Quantitative analysis of relative cell area (*n* = 9). **C** Cell viability assessed by Cell Counting Kit-8 (CCK-8) assay (*n* = 5). **D** Representative Western blot images of the indicated proteins. **E–H** Corresponding densitometric analysis of atrial natriuretic peptide (ANP), brain natriuretic peptide (BNP), β-myosin heavy chain (β-MHC), and HK3 protein levels in AC16 cardiomyocytes. Protein expression was normalized to β-actin (*n* = 3). Data are presented as mean ± SD. Unless otherwise indicated, n denotes independent biological replicates. ∗*P* < 0.05, ∗∗*P* < 0.01, ∗∗∗*P* < 0.001

**Fig. 4:**
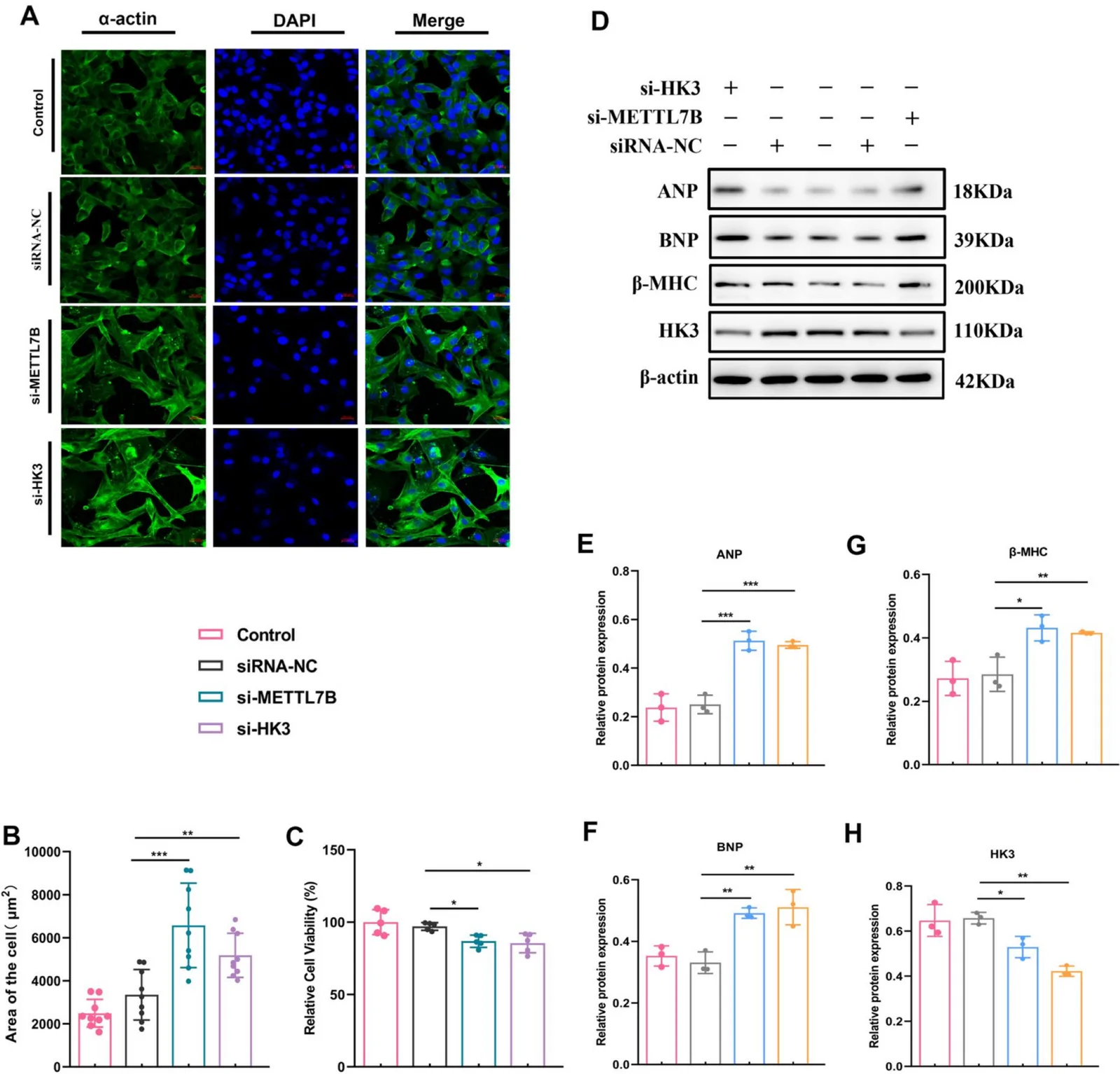
Effects of HK3 or METTL7B silencing on cardiomyocyte hypertrophy and hypertrophy-related marker expression. A Representative immunofluorescence images of AC16 cardiomyocytes stained for α-actinin green, with nuclei counterstained with DAPI blue. Scale bar, 20 μm. **B** Quantitative analysis of relative cell area (*n* = 9). **C** Cell viability assessed by CCK-8 assay (*n* = 5). **D** Representative Western blot images of the indicated proteins. **E–H** Corresponding densitometric analysis of ANP, BNP, β-MHC, and HK3 protein levels in AC16 cardiomyocytes. Protein expression was normalized to β-actin (*n* = 3). Data are presented as mean ± SD. ∗*P* < 0.05, ∗∗*P* < 0.01, ∗∗∗*P* < 0.001

Immunofluorescence analysis revealed that Ang II treatment significantly increased cardiomyocyte size compared with the control group, confirming successful induction of hypertrophy. Overexpression of either METTL7B or HK3 significantly reduced cardiomyocyte enlargement, whereas knockdown of either gene further aggravated the hypertrophic phenotype. Importantly, silencing HK3 in METTL7B-overexpressing cells largely abolished the reduction in cell size conferred by METTL7B Figs. 3A and B and 4A and B, indicating that HK3 may contribute to the anti-hypertrophic effects associated with METTL7B.

Consistent with these findings, CCK-8 assays showed that Ang II significantly reduced cardiomyocyte viability, whereas overexpression of METTL7B or HK3 improved cell viability under hypertrophic stress. In contrast, knockdown of METTL7B or HK3 further impaired viability. Notably, the beneficial effect of METTL7B overexpression was significantly attenuated when HK3 was simultaneously silenced Figs. 3C and 4C.

We next examined hypertrophy-associated molecular markers. The protein levels of ANP, BNP, and β-MHC were markedly elevated following Ang II treatment, confirming the hypertrophic phenotype Fig. 3D-G. Overexpression of METTL7B or HK3 significantly reduced the expression of these markers Fig. 3D-H, whereas knockdown of either gene produced the opposite effect Fig. 4D-G. In addition, METTL7B silencing reduced HK3 protein abundance, further supporting a positive regulatory relationship between the two molecules Fig. 4D and H. More importantly, knockdown of HK3 significantly reversed the suppressive effect of METTL7B overexpression on ANP, BNP, and β-MHC expression Fig. 3D-H. Collectively, these data demonstrate that HK3 functions as a critical downstream effector of METTL7B in protecting cardiomyocytes against hypertrophic remodeling.

### Restoration of METTL7B or HK3 alleviates pressure overload-induced cardiac remodeling in vivo

**Fig. 5:**
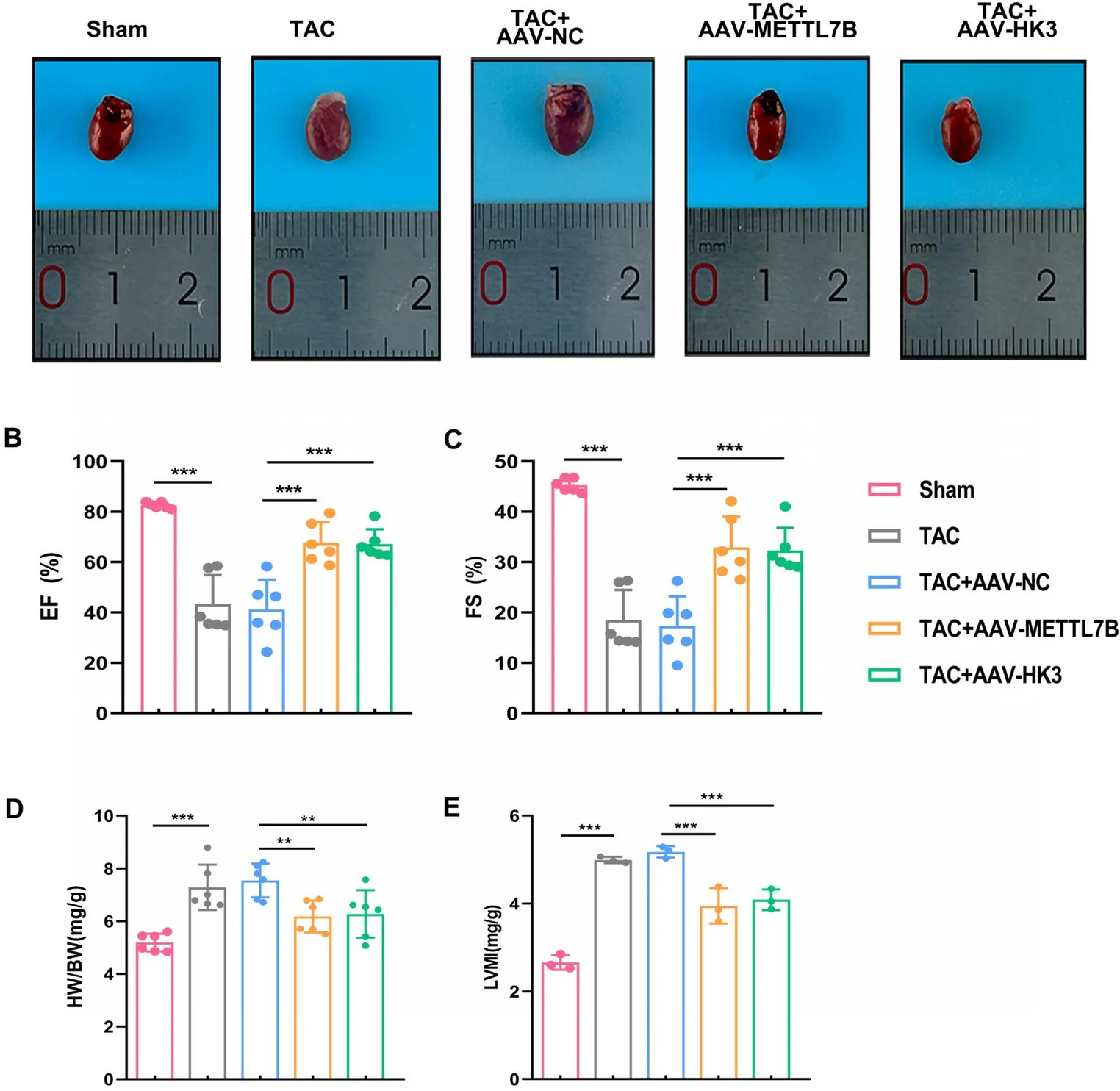
Effects of HK3 or METTL7B overexpression on cardiac morphology and function in mice. **A** Representative gross images of mouse hearts. **B** Echocardiographic assessment of ejection fraction (EF) (*n* = 6 mice per group). **C** Echocardiographic assessment of fractional shortening (FS) (*n* = 6 mice per group). **D** Quantification of the heart weight-to-body weight ratio (HW/BW) (*n* = 6 mice per group). **E** Quantification of left ventricular mass index (LVMI) (*n* = 3 mice per group). Data are presented as mean ± SD. Unless otherwise indicated, n denotes independent biological replicates. ∗*P* < 0.05, ∗∗*P* < 0.01, ∗∗∗*P* < 0.001

To assess the in vivo relevance of the METTL7B-HK3 axis, we generated a TAC-induced mouse model of pathological cardiac hypertrophy and delivered AAV vectors to overexpress METTL7B or HK3. Representative gross images of mouse hearts from each group are shown in Fig. 5A. TAC induced apparent cardiac enlargement compared with the sham group, whereas METTL7B or HK3 overexpression attenuated this gross morphological change. Echocardiographic analysis showed that TAC markedly impaired left ventricular systolic function compared with sham controls, whereas overexpression of either METTL7B or HK3 significantly improved cardiac function Fig. 5B and C. Compared with the TAC plus AAV-NC group, mice overexpressing METTL7B or HK3 exhibited significantly lower HW/BW ratios Fig. 5D. TAC also induced a pronounced increase in LVMI, which was significantly reduced by METTL7B or HK3 overexpression Fig. 5E.

**Fig. 6:**
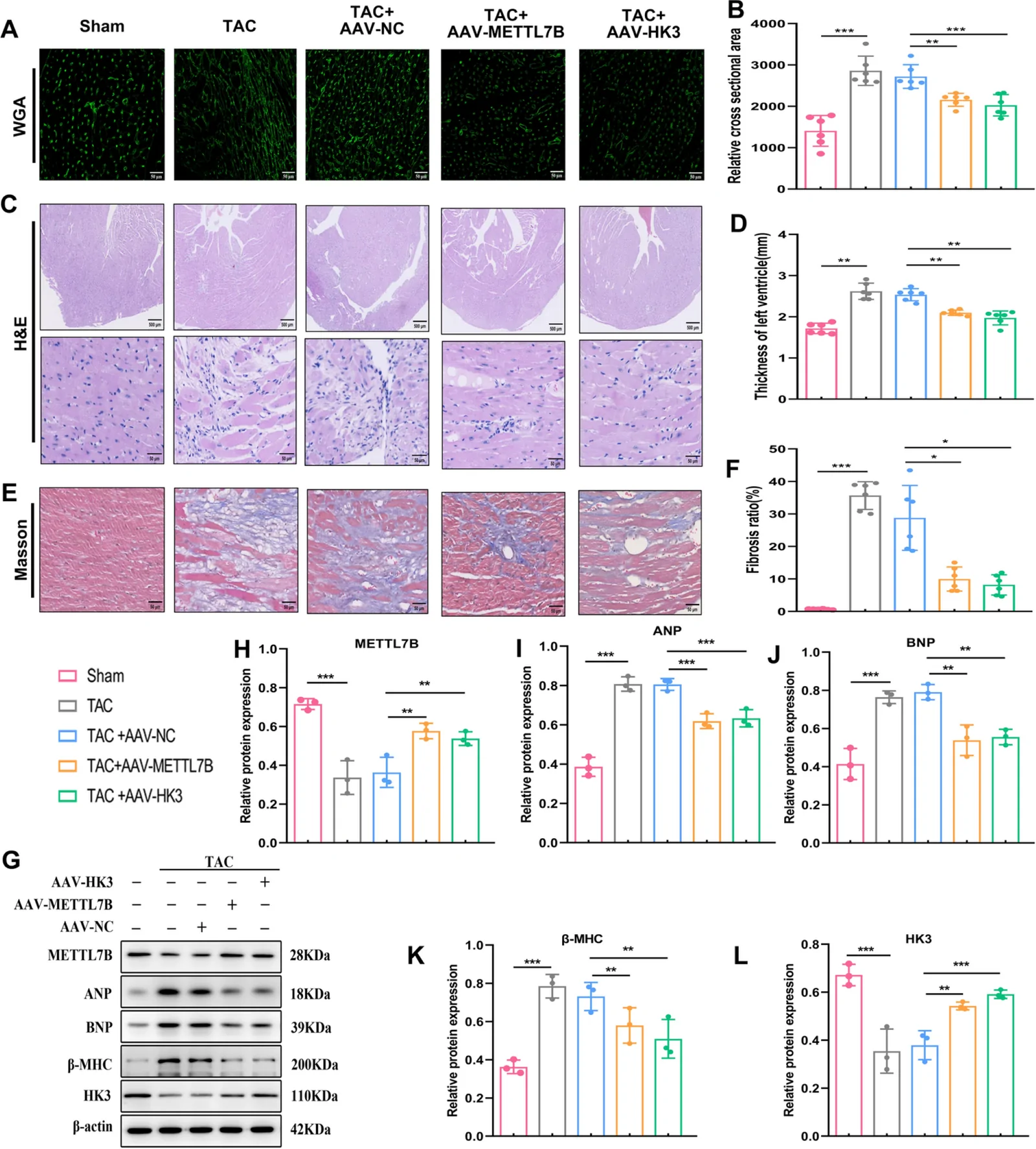
Effects of HK3 or METTL7B overexpression on cardiac remodeling and related protein expression in mice. **A** Representative images of WGA staining in transverse left ventricular myocardial sections. WGA-positive sarcolemmal borders were used to delineate cardiomyocyte profiles. Vascular structures, perivascular regions, interstitial WGA-positive signals, and obliquely or longitudinally sectioned myocytes were excluded from the analysis. Scale bar, 50 μm. **B** Quantification of cardiomyocyte cross-sectional area. Only transversely sectioned cardiomyocytes with intact WGA-positive borders were measured. Each mouse was used as one biological replicate (*n* = 6 mice per group). **C** Representative hematoxylin and eosin (H&E) stained sections of the left ventricular myocardium. Scale bars, 500 μm and 50 μm. **D** Quantification of left ventricular wall thickness (*n* = 6 mice per group). **E** Representative Masson’s trichrome-stained myocardial sections showing collagen deposition in blue. Scale bar, 50 μm. **F** Quantification of fibrotic area (*n* = 6 mice per group). **G** Representative Western blot images of the indicated proteins in mouse left ventricular tissues. **H–L** Densitometric analyses of METTL7B (**H**), ANP (**I**), BNP (**J**), β-MHC (**K**), and HK3 (**L**) protein levels. Protein expression was normalized to β-actin (*n* = 3 mice per group). Data are presented as mean ± SD. Unless otherwise indicated, n denotes independent biological replicates. ∗*P* < 0.05, ∗∗*P* < 0.01, ∗∗∗*P* < 0.001

Morphometric analysis further supported the protective effects of both genes. Histological examination consistently showed attenuation of pathological remodeling in both intervention groups. WGA staining revealed reduced cardiomyocyte cross-sectional area Fig. 6A and B, H&E staining showed amelioration of ventricular wall thickening and myocyte hypertrophy Fig. 6C and D, and Masson staining demonstrated decreased interstitial fibrosis and collagen deposition Fig. 6E and F. These findings indicate that restoration of METTL7B or HK3 effectively suppresses pressure overload-induced myocardial hypertrophy and adverse structural remodeling.

At the molecular level, TAC markedly increased myocardial ANP, BNP, and β-MHC expression, whereas overexpression of METTL7B or HK3 significantly reduced these hypertrophic markers Fig. 6G, I-K. Notably, HK3 overexpression did not alter METTL7B expression in cardiac tissue, whereas METTL7B overexpression significantly increased HK3 expression, supporting the notion that HK3 acts downstream of METTL7B in vivo Fig. 6G, H, L. Taken together, these results validate the cardioprotective role of the METTL7B-HK3 axis in pressure overload-induced cardiac remodeling.

### RNA pull-down suggests YTHDC1 as a candidate HK3 RNA-associated protein, and YTHDC1 depletion stabilizes HK3 mRNA

**Fig. 7:**
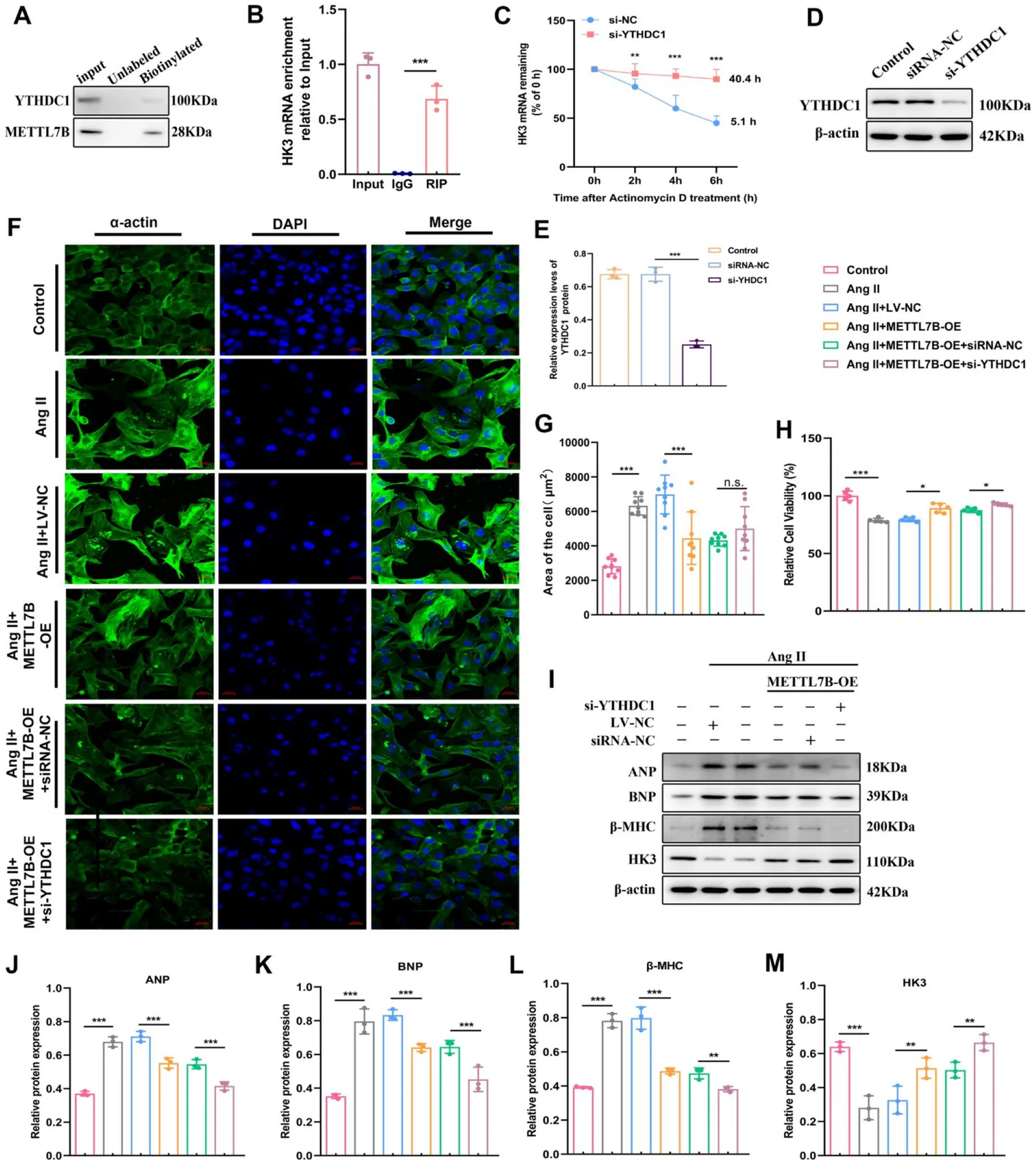
YTHDC1 associates with HK3 mRNA and affects HK3 mRNA stability. **A** RNA pull-down followed by Western blot validation showing enrichment of METTL7B and YTHDC1 in HK3 RNA pull-down complexes. **B** RIP-qPCR was performed using an anti-YTHDC1 antibody and IgG control. HK3 mRNA levels were measured in input, IgG control, and anti-YTHDC1 RIP fractions. HK3 mRNA was significantly enriched in the YTHDC1-RIP fraction compared with the IgG control. Data are shown as mean ± SD from three independent experiments. **C** Actinomycin D chase assay showing HK3 mRNA decay in si-NC and si-YTHDC1 cells. For each biological replicate, HK3 mRNA levels were normalized to the corresponding 0 h level and presented as the percentage of mRNA remaining. The values at the end of each curve indicate the estimated apparent half-life of HK3 mRNA. Data are shown as mean ± SD, *n* = 3. **D**,** E** Representative Western blot images (**D**) and densitometric analysis (**E**) confirming YTHDC1 knockdown in AC16 cardiomyocytes after siRNA transfection. Protein expression was normalized to β-actin (*n* = 3). **F** Representative immunofluorescence images of α-actinin green staining with DAPI blue nuclear counterstaining in AC16 cardiomyocytes. Scale bar, 20 μm. **G** Quantification of relative cardiomyocyte area (*n* = 9). **H** CCK-8 assay of cell viability (*n* = 5). **I** Representative Western blot images of the indicated proteins. **J-M** Densitometric analyses of ANP (**J**), BNP (**K**), β-MHC (**L**), and HK3 (**M**) protein expression. Protein expression was normalized to β-actin (*n* = 3). Data are presented as mean ± SD. Unless otherwise indicated, n denotes independent biological replicates. ∗*P* < 0.05, ∗∗*P* < 0.01, ∗∗∗*P* < 0.001

To explore potential HK3 RNA-associated proteins, we performed RNA pull-down assays followed by protein analysis. LC-MS/MS analysis of proteins recovered from HK3 RNA pull-down eluates initially identified YTHDC1 as a candidate HK3 RNA-associated protein. RNA pull-down followed by Western blotting further validated the enrichment of YTHDC1 in HK3 RNA pull-down complexes and also showed enrichment of METTL7B in these complexes Fig. 7A. To further determine whether YTHDC1 associates with HK3 mRNA in cardiomyocytes, we performed RIP-qPCR in AC16 cells using an anti-YTHDC1 antibody, with IgG as a negative control. HK3 mRNA was significantly enriched in the YTHDC1-RIP fraction compared with the IgG control (*P* < 0.001, Fig. 7B), indicating that HK3 mRNA is present in YTHDC1-containing ribonucleoprotein complexes. These findings suggest that HK3 may be subject to m6A-associated post-transcriptional regulation.

To determine whether YTHDC1 affects HK3 mRNA stability, we performed an Actinomycin D chase assay after YTHDC1 knockdown. After transcriptional inhibition, HK3 mRNA levels in si-NC cells progressively decreased, with approximately 45.0% of HK3 mRNA remaining at 6 h. In contrast, HK3 mRNA decay was markedly attenuated in YTHDC1-depleted cells, with approximately 89.9% of HK3 mRNA remaining at 6 h. Fitting the normalized decay curves to a first-order exponential decay model showed that the apparent half-life of HK3 mRNA increased from approximately 5.1 h in si-NC cells to approximately 40.4 h after YTHDC1 knockdown Fig. 7C. These results indicate that YTHDC1 depletion stabilizes HK3 mRNA, suggesting that YTHDC1 negatively regulates HK3 expression at least in part by affecting HK3 mRNA stability.

To further investigate the potential role of YTHDC1 in HK3 regulation, YTHDC1 was silenced in cardiomyocytes Fig. 7D and E. YTHDC1 knockdown did not produce a significant additional reduction in cardiomyocyte size as assessed by α-actinin staining Fig. 7F and G, but it improved cell viability under hypertrophic stress Fig. 7H. Moreover, YTHDC1 silencing reduced the protein levels of ANP, BNP, and β-MHC and increased HK3 protein abundance in METTL7B-overexpressing cells Fig. 7I-M. These results suggest that YTHDC1 may participate in the post-transcriptional regulation of HK3 under hypertrophic conditions.

## Discussion

In the present study, we identified a previously unrecognized METTL7B-HK3 regulatory relationship and provided evidence that YTHDC1 may participate in the post-transcriptional regulation of HK3 during pathological cardiac hypertrophy. By integrating human transcriptomic and epitranscriptomic datasets with in vitro and in vivo functional analyses, we found that both METTL7B and HK3 were downregulated under hypertrophic conditions, accompanied by reduced m6A enrichment of HK3 transcripts. Functional experiments further showed that restoration of either METTL7B or HK3 attenuated cardiomyocyte hypertrophy in vitro and alleviated pressure overload-induced cardiac remodeling and dysfunction in vivo. In vitro rescue experiments showed that HK3 silencing attenuated the anti-hypertrophic effect of METTL7B overexpression, supporting HK3 as a functional downstream effector of METTL7B in cardiomyocytes. At the molecular level, METTL7B was enriched in HK3 RNA pull-down complexes, and METTL7B overexpression was accompanied by increased m6A enrichment of HK3 transcripts under hypertrophic stress. YTHDC1 was further identified as a candidate HK3 RNA-associated factor that may participate in HK3 post-transcriptional regulation. Together, these findings suggest that METTL7B is linked to HK3 expression and HK3 m6A enrichment, and support HK3 as a functional downstream effector contributing to the anti-hypertrophic effects associated with METTL7B.

Accumulating evidence has established m6A modification as a major post-transcriptional mechanism regulating RNA fate, including RNA splicing, export, translation, and turnover [21, 22]. In the heart, m6A remodeling has emerged as an important determinant of stress adaptation and disease progression [23, 24]. Previous studies have shown that a substantial proportion of cardiac transcripts undergo altered m6A methylation during hypertrophy and heart failure [15], supporting the idea that epitranscriptomic dysregulation is a fundamental feature of pathological remodeling. Moreover, the functional consequences of m6A signaling in the heart appear to be context-dependent. On the one hand, appropriate m6A remodeling may facilitate adaptive responses to hemodynamic stress; on the other hand, sustained or maladaptive changes in m6A machinery can contribute to pathological remodeling and contractile dysfunction [13]. Our findings extend this concept by identifying METTL7B as a hypertrophy-associated m6A-related factor whose reduction is linked to loss of a cardioprotective regulatory relationship associated with HK3 expression.

Most previous work on cardiac m6A regulation has focused on canonical writers such as METTL3 and METTL14. METTL3 has been reported to promote hypertrophic remodeling through several m6A-dependent mechanisms, including regulation of autophagy-related pathways and noncoding RNA processing [13, 18, 25–27], whereas METTL14 has been implicated in exercise-induced physiological hypertrophy and protection against ischemia-reperfusion injury [17]. In contrast, our analysis of human cardiac datasets identified METTL7B, rather than METTL3 or METTL14, as the most significantly altered methyltransferase-like family member in hypertrophic failing hearts. This discrepancy is noteworthy and may reflect differences in disease stage, model system, or clinical context. Unlike experimentally induced early hypertrophy, the human samples analyzed here were derived from patients with established hypertrophy and heart failure, and may therefore better capture epitranscriptomic alterations associated with maladaptive remodeling. Our findings do not necessarily contradict prior work on METTL3 or METTL14, but instead suggest that distinct m6A-related regulators may predominate at different stages of cardiac stress or in different pathological settings.

METTL7B has been reported to possess methyltransferase-related activity and has been implicated in the regulation of oxidative stress, tumor progression, and cellular metabolism [28–33]. However, its role in cardiovascular disease has remained largely unexplored. In this study, we provide evidence that METTL7B exerts a protective effect in the hypertrophic heart. Specifically, METTL7B overexpression restored HK3 expression and was accompanied by increased m6A enrichment of HK3 transcripts, together with suppression of hypertrophic remodeling in cultured cardiomyocytes and TAC mice. In vitro loss-of-function and rescue experiments further showed that HK3 silencing attenuated the anti-hypertrophic effect of METTL7B overexpression, supporting HK3 as a downstream functional effector of METTL7B at the cellular level. Thus, rather than acting as an isolated stress-responsive factor, METTL7B may suppress pathological hypertrophy at least partly through a regulatory relationship involving HK3 as a downstream functional effector.

Among all hexokinase family members, HK3 was uniquely characterized in our dataset by concurrent reduction in transcript abundance and m6A enrichment. This observation is of particular interest because HK3 is a glycolysis-related enzyme that catalyzes the phosphorylation of glucose to glucose-6-phosphate, an early step in glucose utilization [34, 35]. Metabolic remodeling is a hallmark of pathological cardiac hypertrophy, often involving altered substrate utilization, reduced metabolic flexibility, and increased reliance on glucose-associated pathways [36–38]. Although such metabolic reprogramming may initially serve an adaptive role, persistent imbalance can promote energetic inefficiency, fibrosis, and contractile dysfunction. In this context, the identification of HK3 as a functional downstream effector of METTL7B suggests a potential link between m6A-associated post-transcriptional regulation and metabolism-related remodeling in the hypertrophic heart. Notably, METTL7B has also been implicated in the regulation of glycolysis-related programs in other disease settings, including acute lymphoblastic leukemia [39], which supports the biological plausibility of a relationship between METTL7B and metabolic regulation. However, these prior findings do not substitute for direct metabolic measurements in cardiomyocytes. In the present study, our data do not directly demonstrate that METTL7B alters glycolytic activity or cellular energy metabolism in cardiomyocytes. Future studies incorporating direct metabolic assays will be required to determine whether the protective effects associated with METTL7B and HK3 involve direct metabolic regulation, noncanonical survival signaling, or both.

An additional finding of this study is the identification of YTHDC1 as a potential modulator of HK3 regulation. m6A-modified transcripts require reader proteins to translate methylation signals into functional outcomes [40, 41]. YTHDC1, a nuclear m6A reader, has been implicated in alternative splicing, nuclear export, and chromatin-associated RNA regulation [42–44]. In this study, YTHDC1 was enriched in the HK3 RNA pull-down complex, and YTHDC1 knockdown increased HK3 abundance while reducing hypertrophic marker expression in METTL7B-overexpressing cardiomyocytes. Moreover, Actinomycin D chase assays showed that YTHDC1 depletion markedly prolonged the apparent half-life of HK3 mRNA, suggesting that YTHDC1 may negatively regulate HK3 expression, at least in part, by affecting HK3 mRNA stability. However, although RIP-qPCR supports an association between YTHDC1 and HK3 mRNA, the mechanism by which YTHDC1 regulates HK3 remains unclear. As a nuclear m6A reader, YTHDC1 may promote HK3 mRNA turnover by recruiting RNA-processing factors, alter HK3 mRNA stability indirectly through effects on nuclear processing or export, or act together with other m6A-related regulators. Definitive mechanistic elucidation will require CLIP-based mapping of YTHDC1 binding sites, identification of m6A sites on HK3 transcripts, and YTHDC1 mutant rescue experiments.

Several limitations of this study should be acknowledged. First, although METTL7B was enriched in the HK3 RNA pull-down complex and METTL7B overexpression was accompanied by increased m6A enrichment of HK3 transcripts, our data do not demonstrate that METTL7B directly catalyzes m6A modification of HK3 mRNA. Therefore, the current findings should be interpreted as evidence for METTL7B-associated m6A regulation of HK3 rather than definitive proof of direct METTL7B-mediated methylation. Future studies using catalytically impaired METTL7B mutants, in vitro methyltransferase assays, and site-specific m6A mapping will be required to determine whether METTL7B directly modifies HK3 transcripts or indirectly regulates HK3 m6A enrichment. Second, although SRAMP prediction identified multiple putative m6A sites within HK3, including one high-confidence site at position 2220, these results are based on sequence features and should be interpreted as supportive but not definitive evidence. The exact methylated adenosines on HK3 transcripts and the functional relevance of individual methylation motifs were not determined. Therefore, our current data support the presence of potential m6A-associated regulation of HK3, but do not yet establish the functional contribution of individual m6A sites. Further experimental validation, such as site-specific mutagenesis of predicted m6A motifs, SELECT-based m6A detection, or high-resolution m6A mapping approaches, will be required. Third, although our in vitro rescue experiments showed that HK3 knockdown partially counteracted the anti-hypertrophic effect of METTL7B overexpression, these experiments were performed in AC16 cells, which do not fully recapitulate the physiological, structural, and metabolic properties of adult cardiomyocytes. In addition, METTL7B and HK3 double-loss-of-function experiments were not performed, and simultaneous METTL7B overexpression and HK3 suppression was not performed in the TAC model. Therefore, the current data support HK3 as a functional downstream effector of METTL7B at the cellular level, but do not fully establish genetic epistasis or demonstrate that HK3 is required for the cardioprotective effects of METTL7B in vivo. Future studies using more physiologically relevant cardiomyocyte models, together with combined in vivo gain- and loss-of-function approaches will be needed to define the causal role of HK3 in METTL7B-mediated cardioprotection. Fourth, YTHDC1 was identified as an HK3-associated factor, and Actinomycin D chase assays showed that YTHDC1 knockdown prolonged HK3 mRNA half-life. However, the precise mechanism by which YTHDC1 modulates HK3 expression remains to be clarified through additional assays examining RNA binding, RNA processing, nuclear export, mRNA stability, and translational control. Fifth, although WGA staining is widely used to assess cardiomyocyte cross-sectional area, WGA is not cardiomyocyte-specific and can also label vascular, endothelial, and interstitial structures. Therefore, strict morphological criteria were applied, and vascular and interstitial WGA-positive signals were excluded during quantification. Future studies using WGA in combination with cardiomyocyte-specific markers would further improve the precision of cardiomyocyte area measurement. In addition, although HK3 is a glycolysis-related enzyme and our findings suggest a possible link between METTL7B-associated HK3 regulation and metabolism-related remodeling, we did not directly assess metabolic parameters in this study. Glucose uptake, glycolytic flux, lactate production, ATP generation, ECAR/OCR, and isotope-tracing-based metabolic flux analyses were not performed. Therefore, the present data do not establish that METTL7B or HK3 directly alters cardiomyocyte metabolism, and future studies incorporating direct metabolic assays will be required to clarify this possibility. Finally, further validation in independent human myocardial specimens would strengthen the clinical relevance of the METTL7B-HK3 regulatory relationship.

In conclusion, this study identifies METTL7B as a suppressor of pathological cardiac hypertrophy and supports HK3 as a downstream functional effector of METTL7B in cardiomyocytes. METTL7B overexpression was associated with increased HK3 m6A enrichment, restored HK3 expression, and attenuated hypertrophic remodeling in vitro and in vivo. YTHDC1 was further identified as a candidate HK3 RNA-associated factor and known m6A reader whose depletion stabilized HK3 mRNA and increased HK3 abundance, suggesting its potential involvement in HK3 post-transcriptional regulation. These findings expand current understanding of m6A-associated regulation in cardiac hypertrophy and highlight the METTL7B-HK3 regulatory relationship, with YTHDC1 as a potential modulatory factor, as a candidate epitranscriptomic mechanism in pathological cardiac remodeling.

## Supplementary Information

Below is the link to the electronic supplementary material.

Supplementary Material 1

Supplementary Material 2

## Data Availability

No datasets were generated or analysed during the current study.

## Abbreviations

TAC: Transverse aortic constriction
m6A: N6-methyladenosine
Ang II: Angiotensin II
AAV: Adeno-associated virus
PCR: Polymerase chain reaction
NC: Negative control
HW/BW: Heart weight-to-body weight ratio
WGA: Wheat germ agglutinin
H&E: Hematoxylin and eosin
ANP: Atrial natriuretic peptide
BNP: Brain natriuretic peptide
β-MHC: β-myosin heavy chain
siRNAs: Small interfering RNAs
