# Supplementary material for: METTL7B attenuates pathological cardiac hypertrophy via m6A-associated regulation of HK3
Source: Mol Cell Biochem. 2026 Sep 15;481(10):5495–514. doi: 10.1007/s11010-026-05737-1 (PMC13687365; doi:10.1007/s11010-026-05737-1)
Supplement: Supplementary file 1 [file 11010_2026_5737_MOESM1_ESM.docx]

Probe Synthesis: HK3 mRNASequence

>HK3

GCTTTTGGTCATTGGGGTGCCTCATATTGCCAGACAAGAGCTCAGACCTGAGGAGAGTGACTAGCTTCTCTGTGTCCCAGGTGGCCACCTTCCACTGTGGAAGCTCATGGACTCCATTGGGTCTTCAGGGTTGCGGCAGGGGGAAGAAACCCTGAGTTGCTCTGAGGAGGGCTTGCCCGGGCCCTCAGACAGCTCAGAGCTGGTGCAGGAGTGCCTGCAGCAGTTCAAGGTGACAAGGGCACAGCTACAGCAGATCCAAGCCAGCCTCTTGGGTTCCATGGAGCAGGCGCTGAGGGGACAGGCCAGCCCTGCCCCTGCGGTCCGGATGCTGCCTACATACGTGGGGTCCACCCCACATGGCACTGAGCAAGGAGACTTCGTGGTGCTGGAGCTGGGGGCCACAGGGGCCTCACTGCGTGTTTTGTGGGTGACTCTAACTGGCATTGAGGGGCATAGGGTGGAGCCCAGAAGCCAGGAGTTTGTGATCCCCCAAGAGGTGATGCTGGGTGCTGGCCAGCAGCTCTTTGACTTTGCTGCCCACTGCCTGTCTGAGTTCCTGGATGCGCAGCCTGTGAACAAACAGGGTCTGCAGCTTGGCTTCAGCTTCTCTTTCCCTTGTCACCAGACGGGCTTGGACAGGAGCACCCTCATTTCCTGGACCAAAGGTTTTAGGTGCAGTGGTGTGGAAGGCCAGGATGTGGTCCAGCTGCTGAGAGATGCCATTCGGAGGCAGGGGGCCTACAACATCGACGTGGTTGCTGTGGTGAACGACACAGTGGGCACCATGATGGGCTGTGAGCCGGGGGTCAGGCCGTGTGAGGTTGGGCTAGTTGTAGACACGGGCACCAACGCGTGTTACATGGAGGAGGCACGGCATGTGGCAGTGCTGGACGAAGACCGGGGCCGCGTCTGCGTCAGCGTCGAGTGGGGCTCCTTCAGCGATGATGGGGCGCTGGGACCAGTGCTGACCACCTTCGACCATACCCTGGACCATGAGTCCCTGAATCCTGGTGCTCAGAGGTTTGAGAAGATGATCGGAGGCCTGTACCTGGGTGAGCTGGTGCGGCTGGTGCTGGCTCACTTGGCCCGGTGTGGGGTCCTCTTTGGTGGCTGCACCTCCCCTGCCCTGCTGAGCCAAGGCAGCATCCTCCTGGAACACGTGGCTGAGATGGAGGACCCCTCTACTGGGGCAGCCCGTGTCCATGCTATCCTGCAGGACTTGGGCCTGAGCCCTGGGGCTTCGGATGTTGAGCTTGTGCAGCACGTCTGTGCGGCCGTGTGCACGCGGGCTGCCCAGCTCTGTGCTGCCGCCCTGGCCGCTGTTCTCTCCTGCCTCCAGCACAGCCGGGAGCAACAAACACTCCAGGTTGCTGTGGCCACCGGAGGCCGAGTGTGTGAGCGGCACCCCAGGTTCTGCAGCGTCCTGCAGGGGACAGTGATGCTCCTGGCCCCGGAATGCGATGTCTCCTTAATCCCCTCTGTGGATGGTGGTGGCCGGGGAGTGGCGATGGTGACTGCCGTGGCTGCCCGTCTGGCTGCCCACCGGCGCCTGCTGGAGGAGACCCTGGCCCCATTCCGGTTGAACCATGATCAACTGGCTGCGGTTCAGGCACAGATGCGGAAGGCCATGGCCAAGGGGCTCCGAGGGGAGGCCTCCTCCCTTCGCATGCTGCCCACTTTCGTCCGGGCCACCCCTGACGGCAGCGAGCGAGGGGATTTCCTGGCCCTGGACCTCGGGGGCACGAACTTCCGTGTCCTCCTGGTACGTGTGACCACAGGCGTGCAGATCACCAGCGAGATCTACTCCATTCCCGAGACTGTGGCCCAGGGTTCTGGGCAGCAGCTCTTTGACCACATCGTGGACTGCATCGTGGACTTCCAGCAGAAGCAGGGCCTGAGCGGGCAGAGCCTCCCACTGGGTTTTACCTTCTCCTTCCCATGTAGGCAGCTTGGCCTAGACCAGGGCATCCTCCTGAACTGGACCAAGGGTTTCAAGGCATCAGACTGCGAGGGCCAAGATGTCGTGAGTCTGTTGCGGGAAGCCATCACTCGCAGACAGGCAGTGGAGCTGAATGTGGTTGCCATTGTCAATGACACGGTGGGGACCATGATGTCCTGTGGCTATGAGGACCCCCGTTGCGAGATAGGCCTCATTGTCGGAACCGGCACCAATGCCTGCTACATGGAGGAGCTCCGGAATGTGGCGGGCGTGCCTGGGGACTCAGGCCGCATGTGCATCAACATGGAGTGGGGCGCCTTTGGGGACGATGGCTCTCTGGCCATGCTCAGCACCCGCTTTGATGCAAGTGTGGACCAGGCGTCCATCAACCCCGGCAAGCAGAGGTTTGAAAAGATGATCAGCGGCATGTACCTGGGGGAGATCGTCCGCCACATCCTTTTACATTTAACCAGCCTTGGCGTTCTCTTCCGGGGCCAGCAGATCCAGCGCCTTCAGACCAGGGACATCTTCAAGACCAAGTTCCTCTCTGAGATCGAAAGTGACAGCCTGGCCCTGCGGCAGGTCCGAGCCATCCTAGAGGATCTGGGGCTACCCCTGACCTCAGATGACGCCCTGATGGTGCTAGAGGTGTGCCAGGCTGTGTCCCAGAGGGCTGCCCAGCTCTGTGGGGCGGGTGTAGCTGCCGTGGTGGAGAAGATCCGGGAGAACCGGGGCCTGGAAGAGCTGGCAGTGTCTGTGGGGGTGGATGGAACGCTCTACAAGCTGCACCCGCGCTTCTCCAGCCTGGTGGCGGCCACAGTGCGGGAGCTGGCCCCTCGCTGTGTGGTCACGTTCCTGCAGTCAGAGGATGGGTCCGGCAAAGGTGCGGCCCTGGTCACCGCTGTTGCCTGCCGCCTTGCGCAGTTGACTCGTGTCTGAGGAAACCTCCAGGCTGAGGAGGTCTCCGCCGCAGCCTTGCTGGAGCCGGGTCGGGGTCTGCCTGTTTCCCAGCCAGGCCCAGCCACCCAGGACTCCTGGGACATCCCATGTGTGACCCCTCTGCGGCCATTTGGCCTTGCTCCCTGGCTTTCCCTGAGAGAAGTAGCACTCAGGTTAGCAATATATATATATAATTTATTTACA
