# Supplementary material for: METTL7B attenuates pathological cardiac hypertrophy via m6A-associated regulation of HK3
Source: Mol Cell Biochem. 2026 Sep 15;481(10):5495–514. doi: 10.1007/s11010-026-05737-1 (PMC13687365; doi:10.1007/s11010-026-05737-1)
Supplement: Supplementary file 2 [file 11010_2026_5737_MOESM2_ESM.docx]

**Supplementary Figure**

**Fig. S1** **SRAMP-predicted m6A sites in HK3 (NM_002115.3).** Six putative sites were identified at positions 111, 1218, 1863, 1875, 1977, and 2220, with the site at 2220 predicted as high-confidence (combined score 0.658) and the others as moderate-confidence. The upper and lower panels show the score distribution and sequence contexts, respectively.

**Fig. S2 Validation of METTL7B and HK3 knockdown and HK3 overexpression in AC16 cardiomyocytes.** **A** RT-qPCR analysis of METTL7B mRNA levels in AC16 cardiomyocytes transfected with different siRNAs targeting METTL7B. **B** Representative western blot analysis of METTL7B protein expression. **C** Quantification of METTL7B protein levels by densitometric analysis, n = 3. **D** RT-qPCR analysis of HK3 mRNA levels in AC16 cardiomyocytes transfected with different siRNAs targeting HK3. **E** Representative western blot analysis of HK3 protein expression. **F** Quantification of HK3 protein levels by densitometric analysis. **G** RT-qPCR analysis of HK3 mRNA expression after HK3 overexpression in AC16 cardiomyocytes. **H** Representative western blot analysis of HK3 protein expression after HK3 overexpression. **I** Quantification of HK3 protein levels by densitometric analysis. Data are presented as mean ± SD from three independent experiments. ∗*P* < 0.05, ∗∗*P* < 0.01, ∗∗∗*P* < 0.001.
